# On Food

**Published:** 1872-10

**Authors:** 


*On Food ; its varieties, chemical composition, nutritive value, comparative digestibility, etc. Being the substance of four Canton Lectures.* By H. Letheby, M. B., M.A., Ph.D., &c. New York Wm. Wood & Co., 1872. Buffalo : Breed, Lent & Co.

This excellent little work is one intended for general perusal, and will interest the popular reader, looking for information on the subjects of which it treats, as much as the professional man. The history which the author gives of the different varieties of food is written in an attractive and interesting style; in fact the whole work, although some of the tables and statistics are rather dry, is, for a book of its character, presented to the reader in clear, concise and attractive language. The writer seems to live in a state of blissful ignorance concerning American customs and food. On page 16 he says: " There is a sweet and tender variety of Indian Corn called *sugar corn*, the cob or spike of which is cooked entire while in a young, green state, and is either thus served to table as *corn in the cob*, or the grains are separated and served like green peas. This is an American luxury of great delicacy." The author also attributes, (page 17) the " sallow, wheezen look of the natives of the northern States of America," to the " indigestible preparations of Indian Corn, called *mush*, *hominy* or *johnny*," which, he says, " constitute the chief portion of their daily meals."

The first edition met with a ready sale, and the present edition will, no doubt, find many ready purchasers.

